# Luminescence and Magnetic Properties of Two Three-Dimensional Terbium and Dysprosium MOFs Based on Azobenzene-4,4′-Dicarboxylic Linker

**DOI:** 10.3390/polym8020039

**Published:** 2016-02-02

**Authors:** Belén Fernández, Itziar Oyarzabal, José M. Seco, Eider San Sebastián, David Fairen-Jiménez, Santiago Gómez-Ruiz, Alfonso Salinas-Castillo, Antonio J. Calahorro, Antonio Rodríguez-Diéguez

**Affiliations:** 1 Department of Inorganic Chemistry, University of Granada, 18071 Granada, Spain; belenfernandez@ugr.es (B.F.); ajccasanova@ugr.es (A.J.C.); 2 Department of Applied Chemistry, Faculty of Chemistry, University of the Basque Country UPV/EHU, Paseo Manuel Lardizabal 3, 20018 Donostia-San Sebastián, Spain; itziar_oyarzabal@hotmail.com (I.O.); josemanuel.seco@ehu.es (J.M.S.); eider.sansebastian@ehu.es (E.S.S.); 3 Department of Chemical Engineering & Biotechnology, University of Cambridge, Pembroke Street, Cambridge CB2 3RA, UK; df334@cam.ac.uk; 4 Department of Biology and Geology, Physics and Inorganic Chemistry, E.S.C.E.T., Rey Juan Carlos University, c/ Tulipán s/n, 28933 Móstoles, Spain; santiago.gomez@urjc.es; 5 Department of Analytic Chemistry, University of Granada, 18071 Granada, Spain; alfonsos@ugr.es

**Keywords:** metal-organic framework, dysprosium, terbium, magnetism, luminescence

## Abstract

We report the *in situ* formation of two novel metal-organic frameworks based on terbium and dysprosium ions using azobenzene-4,4′-dicarboxylic acid (*H_2_abd*) as ligand, synthesized by soft hydrothermal routes. Both materials show isostructural three-dimensional networks with channels along *a* axis and display intense photoluminescence properties in the solid state at room temperature. Textural properties of the metal-organic frameworks (MOFs) have been fully characterized although no appreciable porosity was obtained. Magnetic properties of these materials were studied, highlighting the dysprosium material displays slightly frequency-dependent out of phase signals when measured under zero external field and under an applied field of 1000 Oe.

## 1. Introduction

Coordination polymers (CPs), also known as metal-organic frameworks (MOFs), have dramatically increased as heterogeneous materials for twenty years. These novel materials have attracted considerable research interest over years as a consequence of their functional properties and potential applications in many scientific areas [1,2]. MOFs are obtained by the self-assembly of metal ions or clusters with appropriate bridging organic ligands. The fact that they can use a variety of metal ions and organic ligands provides the possibility of obtaining a large number of structural motifs generating infinite combinations. The ability to carry out post-synthetic reactions, confer us another synthetic variability to expand the range of targets that we can achieve [3]. The chemistry of Lanthanide-based MOFs has been significantly less explored than that of transition metal-based MOFs, probably due to the fact that lanthanide elements are usually regarded as unsuitable metal centers, as coordination numbers are too high and coordination geometries are hard to control. Over the past few years, however, the design and study of Lanthanide-based MOFs has evolved enormously [4,5,6,7,8,9] because of their interesting structures and potential applications in areas such as luminescent materials [10], selective gas adsorption [11], magnetism [12], sensors, and biological properties [13]. Given that, classically, the first scientific efforts were focused on the use of MOF as possible porous materials for gas storage and separation, the study of their luminescent and magnetic properties have been forgotten. However, increasingly, researchers try to obtain multifunctional MOFs with new interesting physical properties that can give another perspective to MOF’s applications, such as luminescence sensors due to metal-ligand charge transfer [14]. In recent years, we have designed and synthesized novel Lanthanide-MOFs with different nitrogen derivative ligands with interesting luminescent properties [15] and constructed single-molecule magnets (SMM) based on dysprosium, able to show interesting effective energy barriers [16,17]. Lately, we have managed to synthesize Ln-MOFs with SMM behavior, demonstrating the potential of these dicarboxylic linkers to construct new MOFs with novel and interesting properties in this field [18].

**Scheme 1:**
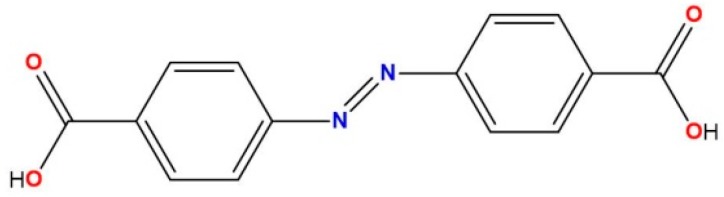
Azobenzene-4,4′-dicarboxylic acid (*H_2_abd*).

Looking among other ligands, the versatile azobenzene-4,4′-dicarboxylate *(abd)*^2−^ linker (Scheme 1) has been used in the preparation of various carboxylate complexes, owing to its rich variety of coordination modes [19]. Moreover, *H_2_abd* is a good candidate to show enhanced emissive properties, which are, in principle, tunable by coordination to metal with different chemical environments. Here, we report the synthesis, structure, luminescence, adsorption and magnetic properties of two isostructural 3D-MOFs {[Tb(abd)_1.5_(H_2_O)_2_(DMF)]∙(H_2_O)_2_∙(DMF)_2_}_n_ (**1**) and {[Dy(abd)_1.5_(H_2_O)_2_(DMF)]∙(H_2_O)_2_∙(DMF)_2_}_n_ (**2**). As we expected, these MOFs are multifunctional materials, particularly the dysprosium material, which shows frequency-dependent out of phase signals when measured under zero external field and under an applied field of 1000 Oe.

## 2. Experimental Section

### 2.1. General Procedures

Unless stated otherwise, all reactions were conducted using hydrothermal conditions. Lanthanide salts and azobenzene-4,4′-dicarboxylic acid were purchased commercially (Sigma-Aldrich, Madrid, Spain) and used without further purification.

### 2.2. Preparation of Complexes

{[Tb(abd)_1.5_(H_2_O)_2_(DMF)]∙(H_2_O)_2_∙(DMF)_2_}*_n_* (**1**): Solvothermal routes were used for the synthesis of this compound. An amount of 0.810 g (3 mmol) of *H_2_abd* were added to 5 mL of DMF and the resulting mixture was sonicated for 10 min. To this solution, 1.21 g (2 mmol) of Tb(CF_3_SO_3_)_3_ in 5 mL of DMF were added and the resulting solution was sealed in a Teflon autoclave and placed into a preheated oven at 95 °C for 48 h. The oven was then opened, and the sealed autoclave was allowed to cool naturally to ambient temperature. The autoclave was opened, the mixture filtered, and the filtrate was allowed to stand at room temperature for several days whereupon orange prismatic crystals of **1** were formed. The crystals were collected by filtration, washed with DMF and dried in a vacuum. Yield: *ca.* 35%; Elemental analysis (%) of C_30_H_41_N_6_O_13_Tb, calcd: C 42.26, H 4.85, N 9.86; found: C 42.64, H 4.62, N 10.18. 

{[Dy(abd)_1.5_(H_2_O)_2_(DMF)]∙(H_2_O)_2_∙(DMF)_2_}*_n_* (**2**): We carried out the same reaction as in the above compound but using Dy(CF_3_SO_3_)_3_ (2 mmol). Orange powder of the compound under study were obtained. Yield: 45%, based on Dy. Anal. Calcd. C_30_H_41_N_6_O_13_Dy: C 42.08, H 4.83, N 9.82. Found: C 42.17, H 4.79, N 10.03.

### 2.3. Physical Measurements

Elemental analyses were carried out at the Centro de Instrumentación Científica (University of Granada) on a Fisons-Carlo Erba analyzer model EA 1108 (Thermo Scientific, Waltham, MA, USA).

### 2.4. Single-Crystal Structure Determination

The crystal structure of **1** was determined by single crystal X-ray crystallography. Suitable crystal of this material was mounted on a glass fibre and used for data collection on a Bruker AXS APEX CCD area detector (Bruker, Billerica, MA, USA) equipped with graphite monochromated MoKα radiation (λ = 0.71073 Å). Lorentz-polarization and empirical absorption corrections were applied [20]. The structures were solved using direct methods and refined with full-matrix least-squares calculations on *F*^2^ using the program SHELX-97 [21]. Anisotropic temperature factors were assigned to all atoms except for hydrogen atoms, which are riding their parent atoms with an isotropic temperature factor, arbitrarily chosen as 1.2 times that of the respective parent. Attempts to solve disorder problems with two crystallization water molecules failed in compound **1**. Instead, a new set of *F*^2^ (hkl) values with the contribution from solvent molecule withdrawn was obtained using the SQUEEZE procedure implemented in PLATON-94 [22]. Final R(F), *wR(F*^2^*)* and goodness of fit agreement factors, details on the data collection and analysis can be found in Table 1. Compounds **1** and **2** are isostructural materials. We carried out a LeBail refinement (Figure S1) with TOPAS [23] software to establish the purity and the unit cell of the powders pertaining to **2**. Cambridge Crystallographic Data Centre (CCDC) reference number for **1** is 1049582. Copies of the data can be obtained free of charge upon application to CCDC, 12 Union Road, Cambridge CB2 1EZ, U.K. (Fax: +44-1223-336-033; www.ccdc.cam.ac.uk/deposit).

**Table 1:** Crystallographic data and structural refinement details for **1**.

Compound	1
Chemical formula	C_30_H_41_N_6_O_13_Tb
M/gmol^−1^	852.61
T(K)	100(2)
λ/Å	0.71073
Crystal system	Triclinic
Space group	*P-1*
a/Å	9.7799(5)
b/Å	11.5661(7)
c/Å	16.7597(10)
α/deg	105.886(2)
β/deg	100.772(2)
γ/deg	100.290(2)
*V*/Å^3^	1,737.46(17)
*Z*	2
ρ_calcd_ (g·cm^−3^)	1.630
μ(mm^−1^)	2.108
*R(int)*	0.0741
GOF on F2	1.026
*R*_1_ *[I > 2σ(I)]*	0.0544
*wR*^2^ *[I > 2σ(I)]*	0.1173

*R*_1_ = Σ||F_0_|–|F_c_||/Σ|F_0_|; *wR*^2^ = [Σ*w*(F_0_^2^ – F_c_^2^)^2^/Σ*w*F_0_^2^]^1/2^.

### 2.5. Luminescence Measurements

A Varian Cary-Eclipse Fluorescence Spectrofluorimeter (Agilent Technologies, Madrid, Spain) was used to obtain the fluorescence spectra. The spectrofluorimeter was equipped with a xenon discharge lamp (peak power equivalent to 75 kW), Czerny-Turner monochromators, R-928 photomultiplier tube, which is red sensitive (even 900 nm), with manual or automatic voltage controlled using the Cary Eclipse software for Windows 95/98/NT system. The photomultiplier detector voltage was 700 V and the instrument excitation and emission slits were set at 5 and 5 nm, respectively.

### 2.6. Computational Calculations

The material was characterized geometrically, starting from the crystallographic coordinates. The pore size distribution (Figure S2) was calculated using the method of Gelb and Gubbins, where the largest sphere that can fit in a random point within a structure without overlapping the van der Waals surface of the framework recorded for a large number of random points [24,25].

### 2.7. Magnetic Measurements

Magnetization and variable-temperature (1.9–300 K) magnetic susceptibility measurements on polycrystalline samples were carried out with a Quantum Design SQUID MPMS XL-5 device (Quantum Design, San Diego, CA, USA) operating at different magnetic fields. The experimental susceptibilities were corrected for the diamagnetism of the constituent atoms by using Pascal’s tables. Ac susceptibility measurements under different applied static fields were performed by using an oscillating ac field of 3.5 Oe on a PPMS 6000 magnetometer.

## 3. Results and Discussion

The hydrothermal reaction of the appropriate lanthanide trifluoromethanesulfonate (2 mmol) with the azobenzene-4,4′-dicarboxylic acid (3 mmol) in dimethylformamide (10 mL) at 95 °C for 48 h produced prismatic orange crystals of these 3D-MOFs. The crystal structure of the terbium compound was determined using single crystal X-ray crystallography.

### 3.1. Description of the Structure

**Figure 1:**
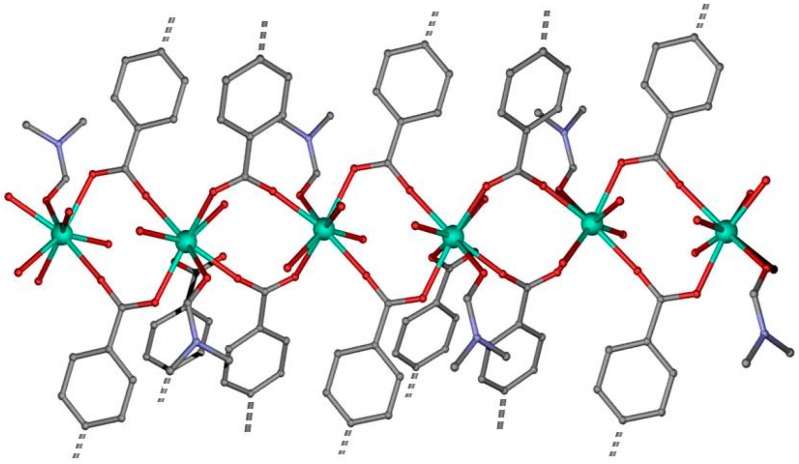
Perspective view of one of the chains that forms the 3D-MOF by double carboxylate-bridge through (*abd)*^2−^ linker.

Compound **1** crystallizes in the triclinic space group *P-1*. The 3D-MOF structure is composed of Tb(III) chains (Figure 1) connected by oxygen atoms pertaining to the carboxylate groups of the (*abd)*^2−^ ligands. In material **1**, the (*abd)*^2−^ linker shows a bis-bridging intradinuclear coordination mode.

**Figure 2:**
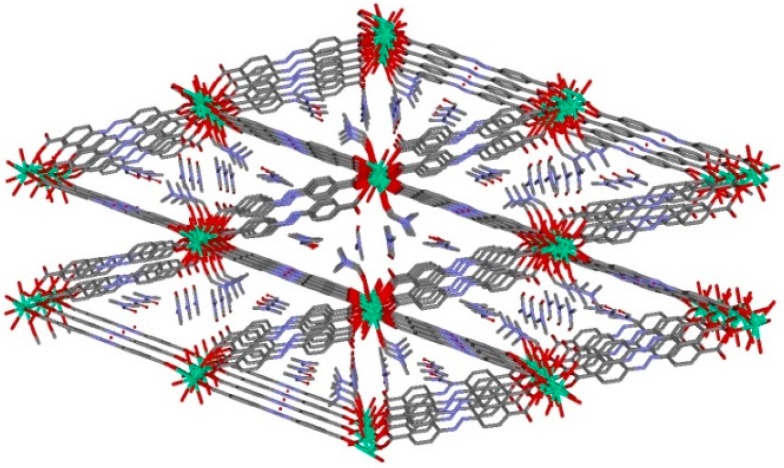
View down a axis of channels in MOF **1**. Hydrogen atoms have been omitted for clarity. Color code N = blue, O = red, C = grey, Tb = green.

The TbO_8_ coordination polyhedron is formed by five oxygen atoms of carboxylate groups pertaining to five different (*abd)*^2^^−^ ligands, two water molecules and one oxygen atom from one DMF coordinated molecule. The Tb-O bond distances are in the range 2.307(4)–2.497 Å, in which the largest corresponds to Tb-O_DMF_ distance. In the terbium chain, the terbium ions are bridged by a double carboxylate-bridge through (*abd)*^2*−*^ linker in which the Tb∙∙∙Tb distance has a value of 4.779 Å, while the shortest interchain Tb∙∙∙Tb distance is of 16.760 Å. As above, this MOF can be described by terbium chains bridged by (*abd)*^2*−*^ linkers generating a three-dimensional network (Figure 2) with channels along *a* axis with DMF solvent molecules inside. These crystallization DMF molecules are involved in strong hydrogen bonds (2.686 Å) with the coordinated water molecule O2W.

The hydrothermal reaction of dysprosium trifluoromethanesulfonate with azobenzene-4,4′-dicarboxylic acid in dimethylformamide produced prismatic orange crystals. The crystal structure of dysprosium material **2** was determined using powder X-ray diffraction. The structure is isostructural to **1**, corroborated by LeBail refinement (Figure S1) implemented in Topas-R.

### 3.2. Adsorption Properties

N_2_ adsorption isotherms in MOF **1** were undertaken at 77 K using a Micromeritics 3Flex instrument. Samples were activated at 393 K for 8 h previous to the adsorption measurement; however, no appreciable porosity was obtained. Following this, we studied the existence of potential changes in the structure during the activation by running a PXRD after the treatment. However, no differences were found before and after the experiment, confirming the no existence of important changes in the structure of the bulk solid. In this context, we and others have found before that the presence of densified layers at the surface of the material may prevent the entry of small molecular species into the bulk porosity [26,27].

### 3.3. Luminescence Studies

**Figure 3:**
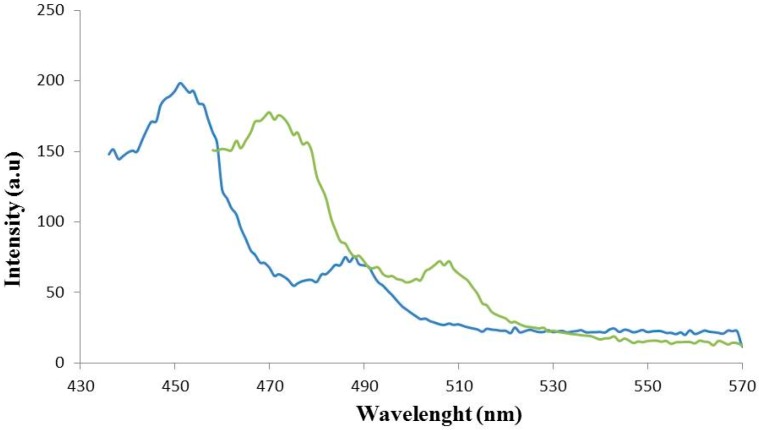
Experimental emission spectra of **1** (blue line) and **2** (green line). Horizontal axis: wavelength (nm); vertical axis: intensity (arbitrary units, a.u.).

Figure 3 shows the emission spectra of **1** and **2** in the solid state at room temperature. Broad intense emission bands are observed for **1**, centred at *ca.* λ = 453 and 488 nm upon excitation at *λ* = 330 nm. The emission of **2**, however, is significantly blue-shifted compared to that of **1**, with similar emission bands centered at *λ* = 475 and 508 nm. The latter shift should be derived from a charge-transfer phenomenon attributed to the breach of a conjugated system of σ and π bonds in the carboxylate groups arisen from the coordination of the ligand and Ln centers, as reported in recent literature in the field [28,29,30].

**Figure 4:**
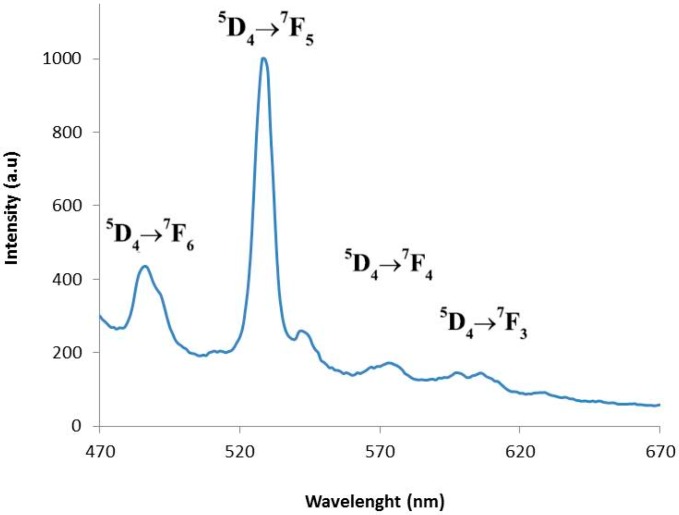
Emission spectrum of terbium MOF.

The luminescence excitation spectrum of **1** was investigated under the typical emission (540 nm) of the terbium(III) ion at room temperature. Figure 4 shows the emission spectrum of this compound at *λ_ex_* = 380 nm. Four characteristic peaks of Tb^3+^ are assigned to the ^5^D_4_→^7^F_J_ transitions, namely, ^5^D_4_→^7^F_6_ (485 nm), ^5^D_4_→^7^F_5_ (538 nm), ^5^D_4_→^7^F_4_ (579 nm), and ^5^D_4_→^7^F_3_ (608 nm), respectively. The most intense emission peak is the hypersensitive transition ^5^D_4_→^7^F_5_ [31,32]. This strong intensity of **1** makes it a desired candidate for luminescence-emitting materials.

### 3.4. Magnetic Properties

**Figure 5:**
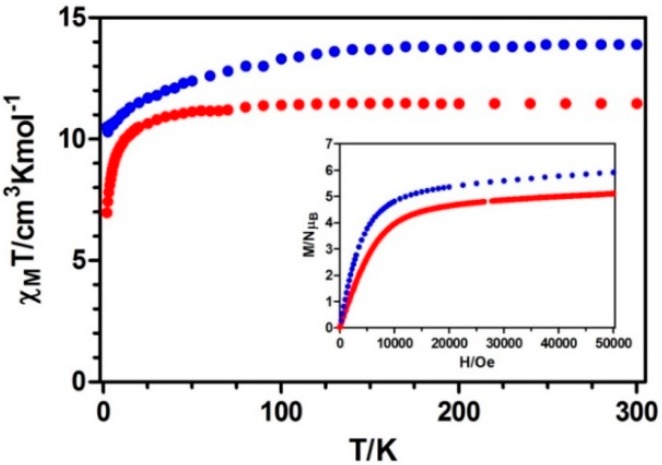
Temperature dependence of the *χ_M_T* product at 1000 Oe for **1** and **2**. Inset: *M versus H* plots for complexes **1** and **2**. Terbium (red) and Dysprosium (blue).

Figure 5 shows the temperature dependences of the magnetic susceptibilities for compounds **1** and **2** measured on polycrystalline samples in the 2–300 K temperature range under an applied field of 0.1 T. The observed *χ_M_T* values at room temperature of 11.46 and 13.90 cm^3^·K·mol^−1^, respectively, for **1** and **2** are close to those expected for independent Ln^III^ ions in the free-ion approximation (11.82 cm^3^·K·mol^−1^ for **1** and 14.17 cm^3^·K·mol^−1^ for **2**).

On lowering the temperature, *χ_M_T* values experience a progressive decrease, as expected for the selective depopulation of the excited Stark sublevels. However, this magnetic response could also involve a significant contribution from antiferromagnetic exchange interactions between lanthanide ions, as anti-*syn* carboxylate bridges are known to mediate both ferro and antiferromagnetic interactions. The linear fit of *χ_M_*^−1^ *vs. T* in the high temperature range obeys the Curie-Weiss law 1/*χ_M_T* = (T − θ)/C, with values of C = 11.47, θ = −0.161 and C = 14.19, θ = −5.831, respectively for **1** and **2** (Figure S3). Due to the presence of strong spin-orbit coupling effects in Tb^3+^ and Dy^3+^ ions, it is difficult to conclude if the small and negative Weiss temperatures indicate antiferromagnetic interactions between adjacent Ln^3+^ ions. The lack of an appropriate model to determine the two contributions in such 3D systems with large anisotropy prevents us from knowing the nature of the magnetic interactions.

**Figure 6:**
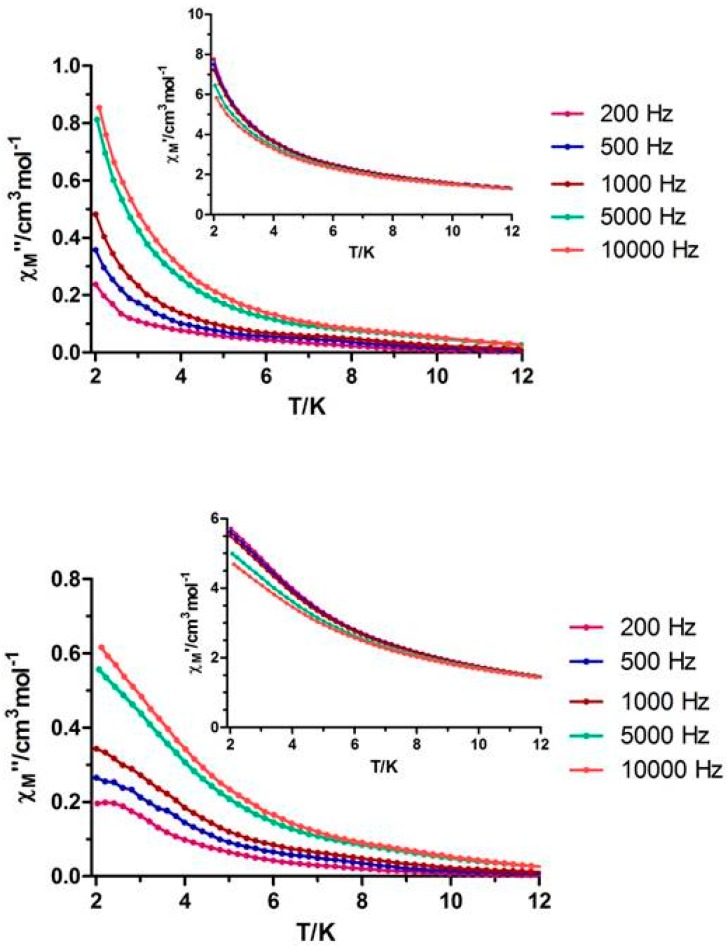
(**Top**) Temperature dependence of in-phase χ*′_M_* (inset) and out-of phase *χ″_M_* components of the alternating current (AC) susceptibility for complex **2** under zero applied external field; (**Bottom**) Temperature dependence of in-phase χ*′_M_* (inset) and out-of phase χ*″_M_* components of the alternating current (AC) susceptibility for complex **2** under an applied field of 1000 Oe.

The field dependence of the molar magnetization at 2 K for **1** and **2** shows a relatively rapid increase at low fields, followed by a very slow increase at high fields without reaching a complete saturation at 5 T. This behavior indicates the presence of magnetic anisotropy and/or the lack of a well-defined ground state, suggesting the presence of low-lying excited states. These low-lying excited states are in agreement with the weak magnetic interactions expected for 4f-4f systems. To further investigate the dynamics of magnetization, we also performed alternating current (AC) magnetic measurements on **1** and **2**. Whereas no out-of-phase (*χ″_M_*) signal was observed for compound **1**, compound **2** displayed slightly frequency-dependent out of phase signals when measured under zero direct current (DC) external field, but without any maxima in the temperature window technically available, which could be due to the quantum tunneling of the magnetization (Figure 6, top).

When the measurements were performed under an applied field of 1000 Oe, no significant improvement was observed, showing only a slight frequency-dependency of the out-of-phase magnetic susceptibility (Figure 6, bottom).

## 4. Conclusions

We have succeeded in the design, synthesis, and characterization of the physical properties of two new MOFs, based on terbium and dysprosium with the interesting azobenzene-4,4′-dicarboxylic acid ligand. These MOFs show isostructural three-dimensional networks and display intense photoluminescence properties in the solid state at room temperature that have been fully characterized. The dysprosium material displays slightly frequency-dependent out-of-phase signals. Therefore, the linkage of luminescence signal and potential magnetic properties of the compounds could signify their utility as multifunctional materials.

## Supplementary Materials

The following are available online at www.mdpi.com/2073-4360/8/2/39/s1. Table S1: Selected bond distances (Å) and Angles (°) 1; Figure S1: Lebail Refinement for **2**: *a =* 9.93, *b =* 11.67, *c =* 16.70, α *=* 106.37, β *= 100.98*, γ *= 100.53*, *V =* 1765.77, *sample displacement =* −0.151 mm; Figure S2: Pore size distribution; Figure S3: Curie-Weiss fit of the *χ_M_^-1^* *vs. T* curves of compounds **1** (top) and **2** (bottom); Figure S4: TGA spectra of MOFs **1** (green) and **2** (blue); Figure S5: UV spectra of compounds 1 (dark blue) and 2 (sky-blue).
