# Supplementary material for: Luminescence and Magnetic Properties of Two Three-Dimensional Terbium and Dysprosium MOFs Based on Azobenzene-4,4′-Dicarboxylic Linker
Source: Polymers (Basel). 2016 Feb 2;8(2):39. doi: 10.3390/polym8020039 (PMC6432568; doi:10.3390/polym8020039)
Supplement: Supplementary file 1 [file polymers-08-00039-s001.pdf]

Supplementary Materials: Luminescence and Magnetic Properties of Two Three-dimensional Terbium and Dysprosium MOFs Based on Azobenzene-4,4′-Dicarboxylic Linker

Belén Fernández, Itziar Oyarzabal, José M. Seco, Eider San Sebastián, David Fairen-Jiménez, Santiago Gómez-Ruiz, Alfonso Salinas-Castillo, Antonio J. Calahorro and Antonio Rodríguez-Diéguez

1. Bond Distances and Angles

Table S1. Selected bond distances (Å) and angles (°) **1**

Bond	Bond distances (Å)	Bond	Bond angles (°)	Bond	Bond angles (°)
Tb1–O1B	2.307(4)	O1B–Tb1–O1A	148.16(14)	O2W–Tb1–O4A	126.79(12)
Tb1–O1A	2.317(3)	O1B–Tb1–O2A	88.23(13)	O1B–Tb1–O3W	78.28(12)
Tb1–O2A	2.358(3)	O1A–Tb1–O2A	98.34(12)	O1A–Tb1–O3W	73.60(13)
Tb1–O2B	2.372(4)	O1B–Tb1–O2B	108.51(13)	O2A–Tb1–O3W	74.68(12)
Tb1–O2W	2.386(4)	O1A–Tb1–O2B	84.99(13)	O2B–Tb1–O3W	140.56(12)
Tb1–O4A	2.407(3)	O2A–Tb1–O2B	142.37(13)	O2W–Tb1–O3W	139.35(12)
Tb1–O3W	2.467(4)	O1B–Tb1–O2W	72.84(13)	O4A–Tb1–O3W	71.57(12)
Tb1–O1C	2.497(4)	O1A–Tb1–O2W	138.99(13)	O1B–Tb1–O1C	143.11(13)
		O2A–Tb1–O2W	76.40(13)	O1A–Tb1–O1C	68.11(13)
		O2B–Tb1–O2W	76.90(13)	O2A–Tb1–O1C	75.40(13)
		O1B–Tb1–O4A	77.20(12)	O2B–Tb1–O1C	71.20(12)
		O1A–Tb1–O4A	79.96(12)	O2W–Tb1–O1C	71.27(13)
		O2A–Tb1–O4A	145.26(13)	O4A–Tb1–O1C	132.86(12)
		O2B–Tb1–O4A	72.34(13)	O3W–Tb1–O1C	126.44(12)

2. LeBail Refinement

Compound **2** is isostructural to **1**. We realized a LeBail refinement (Figure S4) with TOPAS software to establish the purity and the unit cell of the powders pertaining to this material.

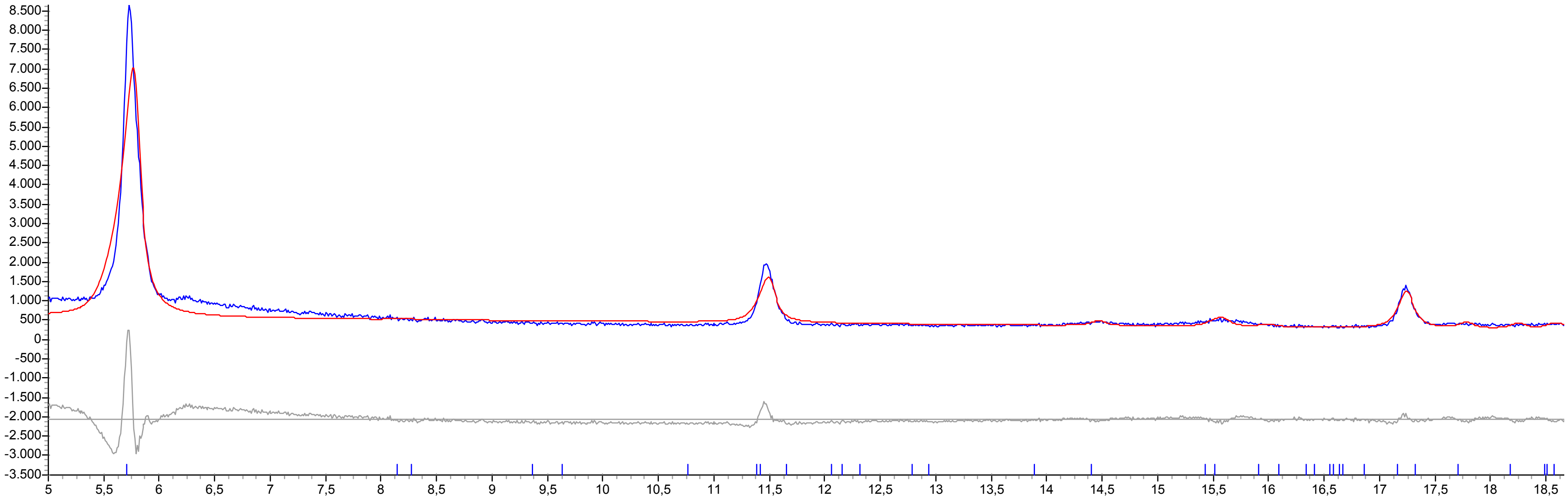

Figure S1. Lebail Refinement for **2**: *a* = 9.93, *b* = 11.67, *c* = 16.70, *α* = 106.37, *β = 100.98*, *γ = 100.53*, *V* = 1765.77, *sample displacement* = −0.151 mm.

3. Pore Size Distribution

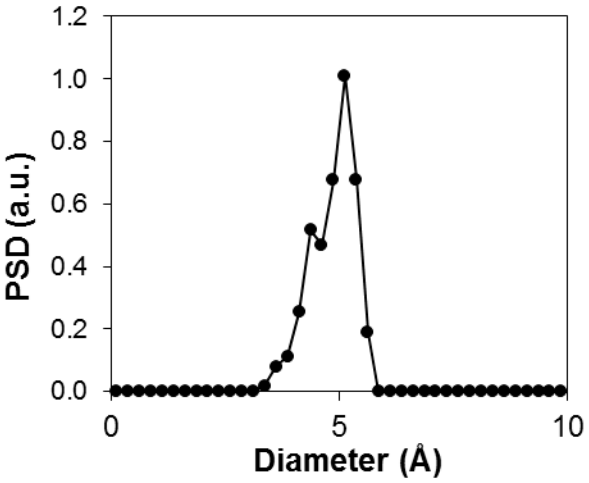

Figure S2. Pore size distribution.

4. Magnetic Properties

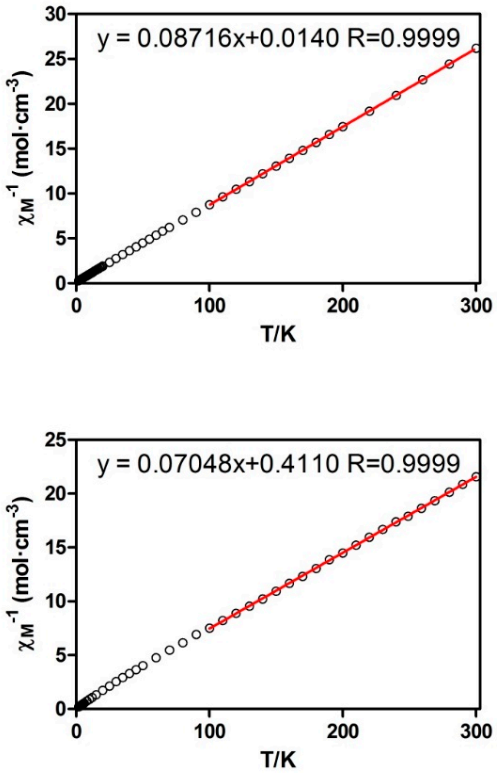

Figure S3. Curie-Weiss fit of the χ_M^{-1} *vs. T* curves of compounds **1** (**top**) and **2** (**bottom**).

5. TGA Spectra

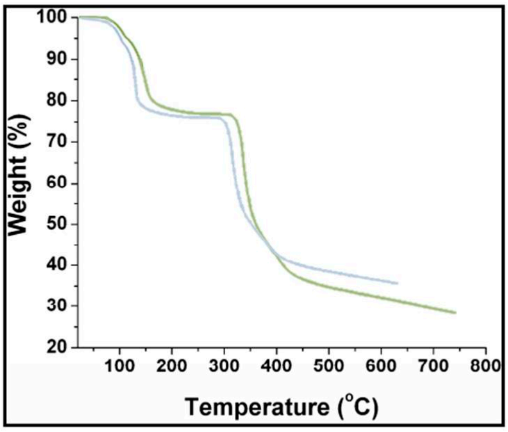

Figure S4. TGA spectra of MOFs **1** (green) and **2** (blue).

6. UV Spectra

Steady-state measurements were performed using a Hewlett Packard diode array spectrophotometer (model 8453; Nortwalk, CT, USA) interfaced to a Pentium MMX 200 microcomputer via an HP IB interface board for absorption measurement.

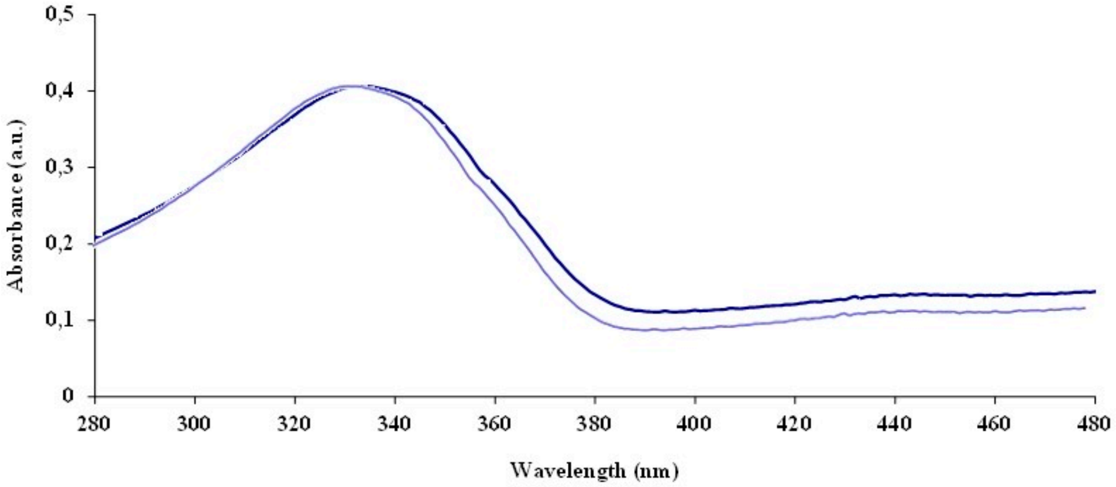

Figure S5. UV spectra of compounds 1 (dark blue) and 2 (sky-blue).

© 2016 by the authors; licensee MDPI, Basel, Switzerland. This article is an open access article distributed under the terms and conditions of the Creative Commons by Attribution (CC-BY) license (http://creativecommons.org/licenses/by/4.0/).
